# Crop Rotation With Cress Increases Cucumber Yields by Regulating the Composition of the Rhizosphere Soil Microbial Community

**DOI:** 10.3389/fmicb.2021.631882

**Published:** 2021-03-12

**Authors:** Xiaoya Gong, Jibo Shi, Xingang Zhou, Tao Yuan, Danmei Gao, Fengzhi Wu

**Affiliations:** ^1^Key Laboratory of Biology and Genetic Improvement of Horticultural Crops (Northeast Region), Ministry of Agriculture and Rural Affairs, Northeast Agricultural University, Harbin, China; ^2^College of Horticulture and Landscape Architecture, Northeast Agricultural University, Harbin, China

**Keywords:** cucumber, rhizosphere soil microbial community, cress rotation, plant–soil feedback, plant–soil microbial interaction

## Abstract

Paddy-upland rotation is an effective agricultural management practice for alleviating soil sickness. However, the effect of varying degrees of flooding on the soil microbial community and crop performance remains unclear. We conducted a pot experiment to determine the effects of two soil water content (SWC) and two flooding durations on the soil microbial community attributes and yield in cucumber. In the pot experiment, cucumber was rotated with cress single (45 days) or double (90 days) under 100 or 80% SWC. Then, the soil microbial were inoculated into sterilized soil to verified the relationship between cucumber growth and microorganisms. The results indicated single cress rotation resulted in a higher cucumber yield than double cress rotation and control. Cress rotation under 80% SWC had higher soil microbial diversity than cress rotation under 100% SWC and control. Flooding duration and SWC led to differences in the structure of soil microbial communities. Under 80% SWC, single cress rotation increased the relative abundance of potentially beneficial microorganisms, including *Roseiflexus* and *Pseudallescheria* spp., in cucumber rhizosphere. Under 100% SWC, single cress rotation increased the relative abundance of potentially beneficial bacteria, such as *Haliangium* spp., and decreased potential pathogenic fungi, such as *Fusarium* and *Monographella* spp., compared with double cress rotation and control. Varying degrees of flooding were causing the difference in diversity, structure and composition of soil microbial communities in the cucumber rhizosphere, which have a positive effect on cucumber growth and development.

## Introduction

Soil sickness caused by continuous monocropping, which often manifests as decreased yields, is mainly the result of soil deterioration from a reduction in the number of key taxa and an accumulation of pathogens in the crop rhizosphere (Banerjee et al., 2019). The structures of microbial communities in the rhizosphere are greatly affected by both fluctuating biotic (e.g., rhizosphere protists) and abiotic (e.g., phenolic acids) factors (Wu et al., 2020; Jin et al., 2020). The application of biocontrol agent (Kohl et al., 2019), chemical induction (Bai et al., 2020), green manuring (Jin et al., 2019) and etc. were used to prevent soil sickness. However, crop rotation is considered the easiest to implement and proven beneficial for alleviating soil sickness (Tiemann et al., 2015).

Paddy-upland rotation is a popular agricultural management practice that involves the sowing of other crops in the gaps between rice plants in an appropriate growing season and reduces the sharing of diseases and pests between the rice and upland crops (Zheng et al., 2016). The rotation system can reduce the amount of greenhouse gas emissions (Xu et al., 2016) and soil pollution (Ni et al., 2021), and more concerns about it are focused on fertilization measures (Alam et al., 2020). Drylands are especially sensitive to the changes in hydrology because flooding drastically affects both above – and underground ecosystem processes (Hou et al., 2017; Feng et al., 2020), such as the changes in the redox potential of the soil following flooding can eliminate toxic substances and soil pathogens (Lee et al., 2020). However, we lack a deep understanding of soil water strategy on dryland. This study will reveal the significance of soil water and its possible impact, and help agriculture predict the possible impact of soil water changes on soil microbial and crop performance.

Water and its availability are important factors affecting the spatial distribution of soil microorganisms (Bahram et al., 2018; Zhou et al., 2018a). The diversity of soil microbial communities increases with increasing soil water content (SWC) in arid and semi-arid ecosystems, but SWC has no major effect on the diversity of soil microbial communities in wetland ecosystems (Cregger et al., 2012; Manzoni et al., 2012). Increasing evidences have fulfilled the gap between the short- and long-term flooding effects on soil microbial communities (Peluola et al., 2013). In environmental ecology, it is observed that flooding has important effects on the underground microbial environment (Shen et al., 2021), but the impact of varying degrees of flooding in agriculture ecology on the crop performance and microbial characteristics is still little known. Understanding the relationships between varying degrees of flooding and soil communities can provide insight into the effects of soil water management strategies on aboveground plant performance and underground microbial communities.

Rhizosphere microbial communities can both sense and affect the health of plants. An important factor affecting plant rhizosphere health is the order of colonization of microorganisms in the rhizosphere; this “priority” effect directly affects plant performance (Toju et al., 2018). The presence of beneficial soil microbial colonies increases plant biomass and interferes with pathogens located on the host through information sensing mechanisms (Turra et al., 2015). Paddy-upland rotation can alter assembly characteristics of microorganisms in the plant rhizosphere, but what these changes affect plants remains unclear.

The continuous cropping of cucumber (*Cucumis sativus* L.) decreases yields (Liu et al., 2020a). Cress [*Oenanthe javanica* (Blume) DC.] can be rotated with cucumber under different water regimes (Chuan and Li, 2019). In this study, we investigated how varying degrees of flooding of cress affected the microbial community in the cucumber rhizosphere. We propose the following hypotheses: (1) soil microbial community attributes will be differentiated according to varying degrees of flooding (2) a longer flooding duration (90 days) and higher water content (100%) have a stronger effect on soil microbial communities in the cucumber rhizosphere; and (3) cress rotation induces changes in the soil microbial community that improve cucumber rhizosphere health and crop growth.

## Materials and Methods

### Cress Rotation Experiment

#### Soil and Plant Preparation

An experiment was conducted in a greenhouse located at the Facilities Horticultural Engineering Center of Northeast Agricultural University, Harbin, China (45°41′N, 126°37′E) in November, 2017. Soil was collected from the upper soil layer (0–20 cm) and sieved (2 mm); cucumber has been planted in the greenhouse since 2006. The soil was sandy loam and contained NH${}_{4}^{+}$-N 25.37 mg⋅kg^–1^, NO${}_{3}^{-}$-N 30.82 mg⋅kg–1, available *P* 109.31 mg⋅kg–1, available K 173.54 mg⋅kg–1, organic carbon 29.89 g⋅kg–1, pH 6.78 (1:2.5, w/v), and electrical conductivity (EC) 0.54 mS⋅cm–1 (1:2.5, w/v).

Cress [*Oenanthe javanica* (Blume) DC, United States.] seeds were provided by Yangzhou University. Before sowing, the seeds were processed by the university to ensure normal germination. The seeds were then evenly spread in a seedling tray. When the cress seedlings reached a height of 10 cm, they were transplanted into pots. Cucumber (*Cucumis sativus* L. cv. Jinzao 1) seeds were soaked in water at 55°C for 30 min and then germinated in a growth incubator at 28°C. Four days later, cucumber seedlings with two cotyledons were used for transplanting.

#### Experimental Design

The experiment was conducted in a pot (upper inner diameter: 23 cm; lower inner diameter: 17 cm; height: 19.5 cm) placed at the same height as the ground and simulating the same environment. Each pot contained 5 kg of soil in which five cress seedlings were cultivated under 100 or 80% SWC. The pots were sealed at the bottom. Rotation with cress was cultivated single (40 days) and double (2 × 40 days: 80 days) until harvest. Three holes were made in the pots with a hole punch when and cucumber was cultivated maintained an SWC of 65%. SWC was detected every 3 days using soil moisture tester (MEET-1000+ soil moisture equipment) provided by Dalian Qifeng Technology Co., Ltd. There were five treatments (two flooding duration, two different SWCs, and one control) in total: (i) cress (80% SWC) – cucumber (SH8); (ii) cress (80% SWC) – cress (80% SWC) – cucumber (SSH8); (iii) cress (100% SWC) – cucumber (SH1); (iv) cress (100% SWC) – cress (100% SWC) – cucumber (SSH1); and (v) continuous cucumber cropping (CK). The experiment was randomly designed with three replicates per treatment and five pots per replication. In total, there were 75 pots (5 treatments × 5 pots × 3 replications).

#### Cucumber Yield and Rhizosphere Collection

Cucumber yield was measured throughout the growing season. After 62 days of cucumber growth.

The cucumber rhizosphere soil from five cucumber plants was mixed into one sample for each replicate and collected using a 2-mm sieve. There were three cucumber rhizosphere soil samples for each treatment. One part of samples was stored at −80°C and used for DNA extraction and used in another experiment aimed at monitoring the effect of changes in the cucumber rhizosphere microbial communities on cucumber seedling growth.

#### DNA Extract, Illumina Miseq Sequencing, and Data Processing

Total soil DNA was extracted from 0.25 g of soil using a Power Soil^®^DNA Isolation Kit (MO BIO Laboratories Inc., Carlsbad, CA, United States) per the manufacturer’s instructions. Each replicate soil sample was extracted in triplicate and the extracted DNA solutions were pooled.

Soil bacterial and fungal community compositions were analyzed using high-throughput amplicon sequencing. Primer sets F338/R806 (Bokulich and Mills, 2013) and ITS1F/ITS2 (Schöler et al., 2017) were used to amplify the V3–V4 regions of the bacterial 16S rRNA gene and the ITS1 regions of the fungal rRNA gene, respectively. Each composite soil sample was independently amplified and the products of the triplicate polymerase chain reaction (PCR) were pooled and purified using 2% agarose gel electrophoresis and an agarose gel DNA purification kit (Agarose Gel DNA Purification Kit, TaKaRa, Mountain View, CA, United States). Next, Tris–HCl precipitation and 2% agarose electrophoresis detection with QuantiFluor^TM^ – ST (Promega Corp., Madison, WI, United States) were used for detection and quantification. The purified amplified fragments were constructed according to the Illumina MiSeq platform (Illumina, San Diego, United States) standard operating procedures to construct PE2 ^∗^ 300 libraries, including (i) connecting the “Y”-shaped connector, (ii) screening to remove linker self-ligated fragments, (iii) enrichment of library templates using PCR amplification, and (iv) sodium hydroxide deformation to produce single-stranded DNA fragments.

The sequenced data were analyzed using Quantitative Insights Into Microbial Ecology (QIIME), Version 1.9.0 software. Before using FLASH software for processing following the methods of Zhou et al. (2017), the original sequence reads were demultiplexed, and quality filtering was carried out to remove low-quality fragments. Finally, FLASH software was used for stitching, and the Cluster Database at High Identity with Tolerance (CD-HIT) was used to perform operational taxonomic unit (OTU) clustering at a similarity level of 97% (Edgar, 2013). Identification and removal of chimeric sequences were carried out using USEARCH 6.1 in QIIME (Zhou et al., 2017). The sequence was normalized effort according to the samples that having minimum sequence number. The bacterial and fungal data sets were deposited in the NCBI-Sequence Read Archive with the submission Accession Numbers SRP269321 and SRP270425, respectively.

### Growth Responses of Cucumber Seedlings to Soil Biota

The experiment was conducted in a sterilized airtight artificial climate chamber. The chamber was maintained at 28°C during the day for 16 h and at 18°C during the night for 8 h, with a light intensity of 21,000 lux. To avoid cross-infection by soil organisms, the environment, cultivated pots, trays, tools, and seeds were all sterilized. Trays were placed under the pots (16 cm diameter, 14 cm height) contained 650 g soils.

#### Soil and Cucumber Seedling Preparation

The field soil was collected from an open field (0–20 cm in depth) of planted wheat using a 1-cm sieve, and called “background soil” as described by Zhou et al. (2017). The “background soil” was autoclaved three times at 121°C and 103 kPa pressure for 30 min (Inderjit, 2006).

Cucumber seeds (cv. Jinzao 1) were sterilized with 2.5% sodium hypochlorite (NaClO) solution for 10 min and then washed thoroughly with distilled water. The seeds were germinated in the dark at 28°C. Fourteen hours later, the germinated cucumber seeds were transferred to sterilized sand. Seven days later, cucumber seedlings with two cotyledons were transplanted. The same sterilization method was used to handle rhizosphere soils from the above rotation experiment (Ndoye et al., 2013). Each pot was used to cultivate one cucumber seedling.

#### Experiment 1: Effects of Soil Sterilization on Cucumber Seedling Growth

The effect of soil microbes on the growth of cucumber seedlings was explored by eliminating soil microbial activity. There were a total of ten treatments: each of the five treatments (CK, SH8, SSH8, SH1, and SSH1) from the above cress rotation experiment was divided into non-sterilized (NS) and sterilized (S) soil treatments. There were three replicates of each treatment and ten pots for each replication. In total, there were 300 pots (10 treatments × 3 replicates × 10 pots).

#### Experiment 2: Effects of Inoculated Soil Biota on Cucumber Seedling Growth

Differently treated soil (6% w/w) (CK, SH8, SSH8, SH1, and SSH1) was mixed with sterilized (S) or not sterilized (NS) background soil (94% w/w) and incubated in the dark for 3 days before transplant the cucumber seedlings (van de Voorde et al., 2012). There were three replicates of each treatment and 10 pots for each replication. In total, there were 300 pots (10 treatments × 3 replicates × 10 pots).

#### Cucumber Seedling Dry Weight Determination

The cucumber seedlings were irrigated with sterile water during the entire cultivation period, and each pot was kept at 50% SWC. The potting position was changed every 3 days. The dry weights of the cucumber seedlings were measured at 10 and 20 days following drying in an oven at 70°C.

### Statistical Analysis

Bacterial and fungal OTUs were compared using the Silva (Release138.2^1^) and Unite 7.2 (Release 8.2^2^) databases. The threshold used was 0.7. We used UCHIME^3^ to identify and delete chimeric sequences. For the Illumina MiSeq sequencing data, alpha diversity was calculated using QIIME (Zhou et al., 2012). For beta diversity, microbial community composition (bacterial and fungal number of OTUs) was analyzed using principal coordinates analysis (PCoA) based on the Bray–Curtis distance dissimilarity, and analysis of similarity (ANOSIM) and multi-response permutation procedure (MRPP) were used to compare microbial community differences between the five treatments (three samples per treatment) with the Bray-Curtis distance and 999 permutations, which were performed in R using the vegan package (Oksanen et al., 2014). The relative abundances of the taxonomic levels of bacterial and fungal phyla and genera between treatments used the “ALDEx2” program package in R to compare and select bacterial and fungal genera (with relative abundances >1% and 0.5% as dominant phyla and genera, respectively) (Fernandes et al., 2014) and analyze differences between rotations with the same/different stubble cress cultivation and the different/same SWCs. The OTUs shared and unique among treatments were counted, and their distributions were illustrated in a Venn diagram with the ***“***Venn Diagram***”*** package in ***“***R***”*** (Yu et al., 2019). Fungal OTUs were assigned to ecological guilds using FUNGuild at the genus and species level (Nguyen et al., 2013). The soil bacterial functional was predicted by PICRUSt from 16S rRNA markers gene sequences on the Galaxy platform, and the biological functions were annotated in the KEGG database (Langille et al., 2013***;*** Kanehisa et al., 2012).

Microsoft Excel (MS Office 2016) was used to organize the raw data. Comparison of the data means between treatments was performed based on the Tukey’s honest significant difference (HSD) test at the 0.05 probability level with IBM SPSS Statistics 27.0 (IBM, Armonk, NY, United States). The drawing of the column charts was carried out using Origin Pro8.5 software.

## Results

### Effect of Rotation With Cress on Cucumber Yield

**FIGURE 1:**
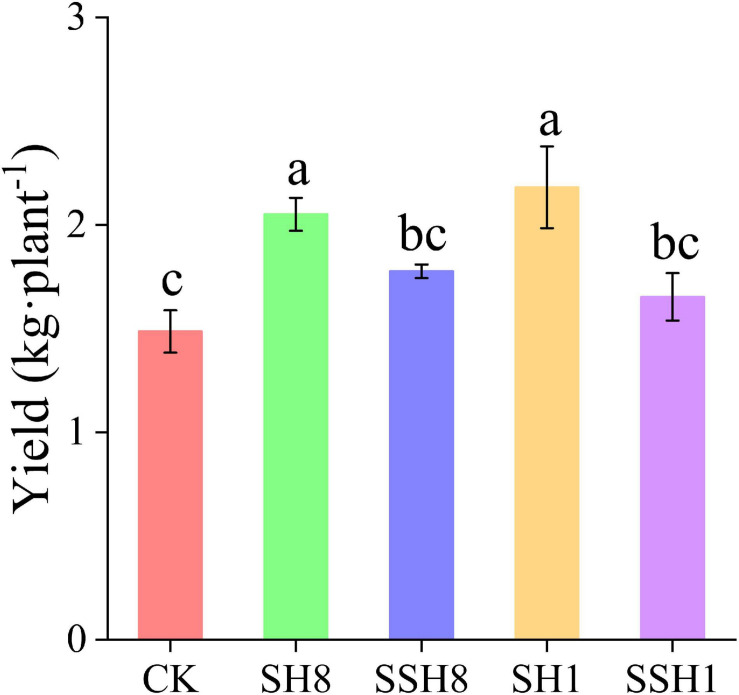
Effect of rotation with cress on cucumber yield. CK represent cucumber continuous cropping, SH8, SSH8, SH1, and SSH1 represent single or double cress rotation under 80% or 100% soil water content. Different letters are significantly different (*P* < 0.05, Tukey’s HSD test).

Cress rotation under 80% SWC and single cress rotation under 100% SWC increased the cucumber yield significantly compared with the control (*P* < 0.05). Single cress rotation also increased the yield significantly compared with double cress rotation (Figure 1) (*P* < 0.05).

### Alpha and Beta Diversities of Bacterial and Fungal Communities in Cucumber Rhizosphere

**FIGURE 2:**
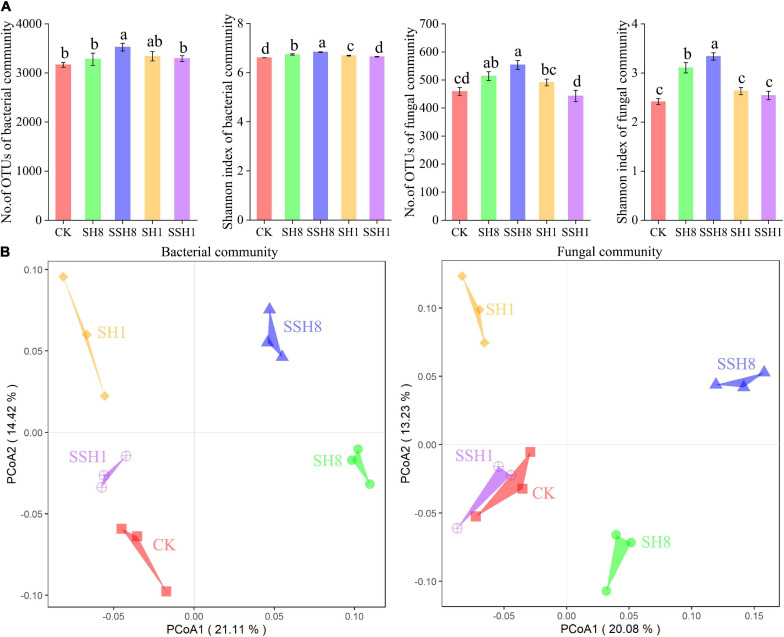
Alpha **(A)** and beta diversities **(B)** of cucumber rhizosphere bacterial and fungal communities. Beta diversities based on the Bray-Curtis distance dissimilarity were visualized by principal component analyses. OTUs were delineated at 97% sequence similarity. Different letters indicate statistically significant differences among treatments (*P* < 0.05, Tukey’s HSD test). CK represent cucumber continuous cropping, SH8, SSH8, SH1, and SSH1 represent single or double cress rotation under 80 or 100% soil water content.

The α-diversities of bacteria and fungi community were significantly higher in cress rotation under 80% SWC than in the control and cress rotation under 100% SWC (*P* < 0.05) (Figure 2A and Supplementary Table 1). However, there is no difference between the control and double cress rotation under 100% SWC (Figure 2A and Supplementary Table 1).

For both the bacterial and fungal communities, PCoA revealed that samples from the same treatment were grouped together, and samples from the five treatments could be clearly distinguished (Figure 2B). Non-parametric multivariate statistical test analyses demonstrated that the compositions of the bacterial and fungal communities in the cucumber rhizosphere differed among treatments (bacterial community: ANOSIM, *R* = 0.68, *P* = 0.001; MRPP, Delta = 0.21, *P* = 0.003; fungal community: ANOSIM, *R* = 0.69, *P* = 0.001; MRPP, Delta = 0.21, *P* = 0.001).

### Compositions of Bacterial and Fungal Communities in Cucumber Rhizosphere

Miseq sequencing data was classified at the 97% similarity level included forty bacterial phyla and five fungal phyla.

**FIGURE 3:**
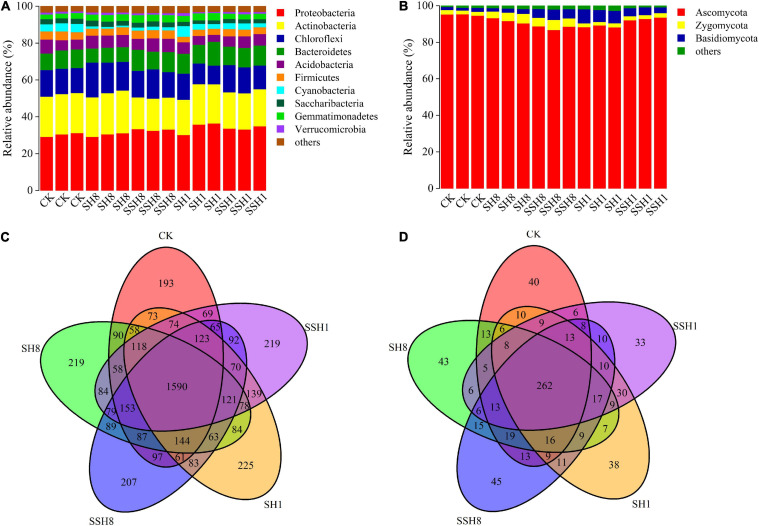
Relative abundances of main bacterial **(A)** and fungal **(B)** phyla and Venn diagram analyses of bacterial **(C)** and fungal **(D)** in cucumber rhizosphere soils treated with different treatments. Bacterial and fungal phyla with average relative abundance>1% were shown and does not contain unclassified taxa. Data are represented as the means of three independent replicates. Venn diagram **(C,D)** demonstrated the numbers of shared and unique observed OTUs at 97% similarity among treatments.

For the bacterial phylum community, double cress rotation under 80% SWC showed a lower relative abundance of Actinobacteria and a higher relative abundance of Firmicutes than single cress rotation under 80% SWC, and double cress rotation had a significantly lower relative abundance of Actinobacteria than the control. Single cress rotation under 80% SWC had a significantly lower relative abundance of Bacteroidetes than the other rotation treatments. Single rotation with cress had a higher relative abundance of Gemmatimonadetes than the control and cress rotation under 80% SWC had a lower relative abundance of Cyanobacteria than the control (*P* < 0.05) (Figure 3A).

For the fungal phylum community, single cress rotation under 80% SWC had a lower relative abundance of Ascomycota than double rotation with cress under 80% SWC and single rotation with cress under 100% SWC, and the control had the highest relative abundance. Basidiomycota had a higher relative abundance in single cress rotation than in double under 100% SWC. Cress rotation under 80% SWC had a higher relative abundance of Zygomycota compared with cress rotation under 100% SWC and the control (*P* < 0.05) (Figure 3B).

Among all samples, single cress rotation under 100% had the highest number of unique OTUs (225) and the control had the lowest number of unique OTUs (193) in bacterial (Figure 3C). Double cress rotation under 80% had the highest number of unique OTUs (45) and the double cress rotation under 100% had the lowest number of unique OTUs (33) in fungal (Figure 3D).

Both single and double cress rotation under 80% SWC had lower relative abundances of *Kribbella* and *Mycobacterium* spp. than the control, and both single and double rotation with cress had higher relative abundances of *Roseiflexus* and *Nitrospira* spp., respectively, under 80% SWC. Moreover, single cress rotation under 100% SWC had higher relative abundances of *Pseudoduganella* spp. than the control. The relative abundance of *Streptomyces*, *Niastella*, *Cellvibrio*, *Fluviicola* and *Clostridium-sensu-stricto-1* spp. were higher in 80% SWC than in 100% SWC (*P* < 0.05) (Table 1).

**TABLE 1:** Relative abundances of main bacterial genus in cucumber rhizosphere soils.

	**CK**	**SH8**	**SSH8**	**SH1**	**SSH1**
*Sphingomonas*	2.88 ± 0.19^a^	3.25 ± 0.13^a^	3.00 ± 0.08^a^	2.85 ± 0.3^a^	3.26 ± 0.25^a^
*Streptomyces*	2.92 ± 0.18^a^	2.04 ± 0.03^b^	1.61 ± 0.15^c^	2.74 ± 0.15^a^	2.82 ± 0.10^a^
*Rhizobium*	2.22 ± 0.03^b^	1.74 ± 0.14^d^	2.15 ± 0.12^b,c^	1.80 ± 0.20^c,d^	2.63 ± 0.14^a^
*Bacillus*	1.34 ± 0.09^a,b^	1.03 ± 0.08^c^	1.53 ± 0.03^a^	0.93 ± 0.09^c^	1.29 ± 0.07^b^
*Niastella*	1.09 ± 0.04^b,c^	0.95 ± 0.01^c^	0.98 ± 0.07^c^	1.20 ± 0.06^b^	1.46 ± 0.08^a^
*Ensifer*	1.08 ± 0.06^a,b^	0.83 ± 0.08^c^	1.07 ± 0.02^a,b^	0.92 ± 0.11^b,c^	1.23 ± 0.11^a^
*Gaiella*	1.03 ± 0.04^a^	1.10 ± 0.17^a^	0.86 ± 0.05^a^	1.08 ± 0.13^a^	0.89 ± 0.03^a^
*Lysobacter*	1.04 ± 0.04^a,b^	0.88 ± 0.06^b^	1.05 ± 0.08^a,b^	1.12 ± 0.15^a^	0.86 ± 0.04^b^
*Devosia*	0.97 ± 0.08^b^	0.81 ± 0.02^b^	0.85 ± 0.02^b^	0.98 ± 0.14^a,b^	1.24 ± 0.15^a^
*Nocardioides*	0.92 ± 0.03^a^	1.16 ± 0.10^a^	0.66 ± 0.02^a^	1.14 ± 0.51^a^	0.90 ± 0.02^a^
*Kribbella*	1.07 ± 0.02^a^	0.91 ± 0.04^b^	0.73 ± 0.05^c^	0.70 ± 0.10^c^	1.01 ± 0.06^a,b^
*Clostridium_sensu_stricto_1*	0.92 ± 0.09^a^	0.91 ± 0.06^a,b^	0.92 ± 0.03^a^	0.71 ± 0.07^c^	0.73 ± 0.07^b,c^
*Amycolatopsis*	1.10 ± 0.15^a^	0.92 ± 0.02^a,b^	0.65 ± 0.05^c^	0.79 ± 0.08^b,c^	0.70 ± 0.03^b,c^
*Pseudarthrobacter*	0.83 ± 0.08^a^	1.13 ± 0.32^a^	0.83 ± 0.04^a^	0.60 ± 0.20^a^	0.78 ± 0.21^a^
*Chitinophaga*	1.05 ± 0.13^a^	0.67 ± 0.04^b^	0.72 ± 0.02^b^	0.94 ± 0.05^a^	0.74 ± 0.04^b^
*Chryseolinea*	0.62 ± 0.05^a^	0.90 ± 0.06^a^	0.77 ± 0.07^a^	0.98 ± 0.50^a^	0.72 ± 0.05^a^
*Flavobacterium*	1.04 ± 0.21^a^	0.43 ± 0.05^c^	0.87 ± 0.02^a,b^	0.85 ± 0.02^a,b^	0.71 ± 0.04^b^
*Steroidobacter*	0.72 ± 0.06^a^	0.77 ± 0.11^a^	0.81 ± 0.05^a^	0.76 ± 0.09^a^	0.80 ± 0.05^a^
*Mycobacterium*	0.81 ± 0.01^a^	0.65 ± 0.07^b,c^	0.64 ± 0.06^b,c^	0.57 ± 0.04^c^	0.77 ± 0.04^ab^
*Pseudoxanthomonas*	0.49 ± 0.05^b^	0.52 ± 0.07^b^	0.79 ± 0.06^a^	0.76 ± 0.07^a^	0.87 ± 0.05^a^
*Aeromicrobium*	0.74 ± 0.03^a^	0.75 ± 0.12^a^	0.49 ± 0.08^b^	0.59 ± 0.02^a,b^	0.58 ± 0.06^a,b^
*Roseiflexus*	0.63 ± 0.04^b^	0.75 ± 0.07^a^	0.62 ± 0.01^b^	0.59 ± 0.04^b^	0.58 ± 0.03^b^
*Taibaiella*	0.50 ± 0.04^c^	0.34 ± 0.02^d^	0.55 ± 0.05^c^	0.78 ± 0.04^b^	0.92 ± 0.05^a^
*Haliangium*	0.49 ± 0.05^d^	0.53 ± 0.03^c,d^	0.67 ± 0.02^a,b^	0.77 ± 0.05^a^	0.59 ± 0.04^b,c^
*Pseudomonas*	0.58 ± 0.08^b^	0.36 ± 0.02^c^	0.83 ± 0.04^a^	0.56 ± 0.04^b^	0.68 ± 0.07^a,b^
*Nitrospira*	0.54 ± 0.07^b,c^	0.57 ± 0.03^b,c^	0.72 ± 0.07^a^	0.64 ± 0.02^a,b^	0.47 ± 0.05^c^
*Cellvibrio*	0.62 ± 0.07 ab	0.40 ± 0.08^b^	0.42 ± 0.07^b^	0.68 ± 0.07^a^	0.70 ± 0.11^a^
*Actinoplanes*	0.45 ± 0.03^b^	0.49 ± 0.03^b^	0.48 ± 0.03^b^	0.57 ± 0.08^b^	0.72 ± 0.03^a^
*Pseudoduganella*	0.54 ± 0.03^b^	0.39 ± 0.06^c^	0.45 ± 0.04^b,c^	0.70 ± 0.06^a^	0.47 ± 0.04^b,c^
*Ohtaekwangia*	0.62 ± 0.04^a^	0.33 ± 0.04^a^	0.42 ± 0.10^a^	0.68 ± 0.33^a^	0.45 ± 0.03^a^
*Fluviicola*	0.41 ± 0.06^c^	0.22 ± 0.03^d^	0.35 ± 0.05^c,d^	0.66 ± 0.01^b^	0.84 ± 0.07^a^

*Values (mean ± standard error) in the same row followed by different letters are significantly different at the 0.05 probability level according to Tukey’s HSD test. Bacterial genus with average relative abundance >0.5% were shown and does not contain unclassified taxa. Different letters are significantly different (*P* < 0.05, Tukey’s HSD test).*

At the genus level, 243 fungal taxa were detected. *Ilyonectria* spp. was higher in both the single and double cress rotation under 80% and 100% SWC than in the control. *Pseudallescheria*, *Mortierella*, *Pseudaleuria*, *Cryptococcus*, and *Mycothermus* spp. were higher under 80% SWC than in the control. *Fusarium* and *Monographella* spp. were lower in the singly rotated cress under 100% SWC than in the control. *Pseudallescheria* spp. had the highest relative abundance in single cress rotation than in other treatments (*P* < 0.05) (Table 2).

**TABLE 2:** Relative abundances of main fungal genus in cucumber rhizosphere soils.

	**CK**	**SH8**	**SSH8**	**SH1**	**SSH1**
*Monosporascus*	45.99 ± 2.06^a^	17.27 ± 0.72^d^	21.68 ± 1.30^c^	43.86 ± 0.97^a^	35.54 ± 1.46^b^
*Fusarium*	14.04 ± 0.44^c^	17.64 ± 0.76^b^	13.50 ± 0.80^c^	10.28 ± 0.43^d^	24.26 ± 1.63^a^
*Chaetomium*	11.66 ± 1.52^b,c^	17.82 ± 1.11^a^	13.68 ± 0.91^b^	11.25 ± 0.09^b,c^	10.47 ± 0.81^c^
*Pseudallescheria*	7.89 ± 0.01^c^	14.88 ± 1.00^a^	12.18 ± 0.58^b^	8.47 ± 0.38^c^	8.78 ± 0.86^c^
*Mortierella*	1.94 ± 0.16^b^	4.09 ± 0.74^a^	4.47 ± 0.39^a^	1.84 ± 0.16^b^	2.09 ± 0.16^b^
*Humicola*	1.99 ± 0.48^b^	3.54 ± 0.08^a^	3.63 ± 0.22^a^	1.73 ± 0.09^b^	2.15 ± 0.24^b^
*Aspergillus*	1.76 ± 0.03^b^	2.49 ± 0.30^a^	1.29 ± 0.14^c^	1.45 ± 0.06^b,c^	1.28 ± 0.05^c^
*Pseudaleuria*	0.54 ± 0.14^c^	1.77 ± 0.24^a^	1.06 ± 0.15^b^	0.57 ± 0.08^c^	0.50 ± 0.13^c^
*Acremonium*	0.57 ± 0.05^c^	0.95 ± 0.05^b^	1.32 ± 0.06^a^	0.60 ± 0.09^c^	0.90 ± 0.08^b^
*Monographella*	0.92 ± 0.09^a^	0.86 ± 0.002^a^	0.88 ± 0.07^a^	0.35 ± 0.02^b^	0.77 ± 0.18^a^
*Clitopilus*	0.01 ± 0.003^c^	0.13 ± 0.01^c^	0.78 ± 0.07^b^	1.51 ± 0.14^a^	1.33 ± 0.49^a,b^
*Cephaliophora*	0.37 ± 0.19b^c^	0.68 ± 0.14^b^	1.92 ± 0.17^a^	0.36 ± 0.06^b,c^	0.16 ± 0.01^c^
*Cladosporium*	0.19 ± 0.04^b^	0.55 ± 0.11^b^	1.96 ± 0.36^a^	0.22 ± 0.03^b^	0.38 ± 0.07^b^
*Olpidium*	0.36 ± 0.16^b^	0.28 ± 0.08^b^	0.23 ± 0.02^b^	1.58 ± 0.37^a^	0.40 ± 0.24^b^
*Kernia*	0.45 ± 0.08^a^	0.62 ± 0.04^a^	0.66 ± 0.05^a^	0.43 ± 0.01^b^	0.31 ± 0.04^b^
*Gibberella*	0.46 ± 0.08^a^	0.54 ± 0.03^a^	0.48 ± 0.01^a^	0.55 ± 0.04^a^	0.43 ± 0.07^a^
*Penicillium*	0.47 ± 0.05^b^	0.46 ± 0.05^b^	0.72 ± 0.11^a^	0.36 ± 0.05^b^	0.37 ± 0.03^b^
*Conocybe*	0.35 ± 0.15^b,c^	0.15 ± 0.04c	0.65 ± 0.08ab	0.90 ± 0.17a	0.22 ± 0.02^c^
*Thermomyces*	0.31 ± 0.01^b^	0.79 ± 0.04^a^	0.42 ± 0.07^b^	0.33 ± 0.02^b^	0.32 ± 0.06^b^
*Remersonia*	0.31 ± 0.03^b^	0.61 ± 0.03^a^	0.61 ± 0.04^a^	0.32 ± 0.02^b^	0.33 ± 0.01^b^
*Cryptococcus*	0.25 ± 0.05^b^	0.57 ± 0.08^a^	0.53 ± 0.08^a^	0.30 ± 0.04^b^	0.16 ± 0.03^b^
*Thanatephorus*	0.30 ± 0.10^a,b^	0.04 ± 0.01^b^	0.12 ± 0.02^b^	0.69 ± 0.13^a^	0.65 ± 0.33^a^
*Mycothermus*	0.27 ± 0.06^b,c^	0.53 ± 0.02^a^	0.43 ± 0.02^a^	0.32 ± 0.03^b^	0.21 ± 0.04^c^

*Different letters are significantly different (*P* < 0.05, Tukey’s HSD test).*

#### Microbial Ecological Guilds in the Cucumber Rhizosphere

**FIGURE 4:**
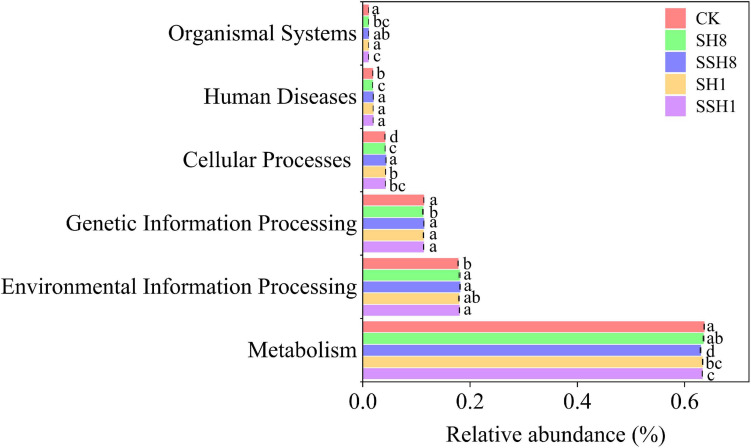
Soil bacterial function prediction of cucumber rhizosphere in different treatments (Hierarchy level 1).

There are six types of primary functional layers, including metabolism, environmental information processing, genetic information processing, cellular processes, human diseases, and organismal systems (Figure 4), and 19 secondary functional layers (Supplementary Table 2).

In the primary metabolic pathway, the relative abundance of metabolism was significantly lower in the single cress rotation under 100% SWC and the double cress rotation cress than in the control (*P* < 0.05) (Figure 4). Metabolism of terpenoids and polyketides and lipid metabolism were lower in the double cress rotation than in the control. Nucleotide metabolism and metabolism of cofactors and vitamins were lower in the single cress rotation than in the control (*P* < 0.05) (Supplementary Table 2).

**FIGURE 5:**
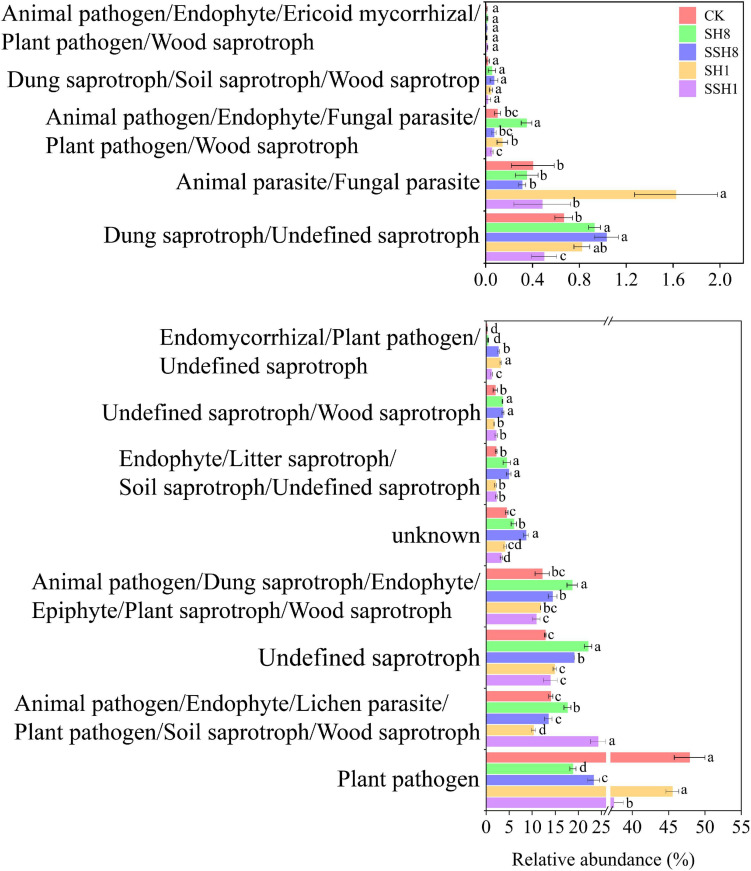
Effect of cress rotation on the relative abundance of fungal ecological functional in cucumber rhizosphere soil.

A total of 251 out of 300 fungal OTUs (83%) were used for function prediction. Cress rotation changed the fungal ecological function in the cucumber rhizosphere. Most taxa were classified as plant pathogens, and plant pathogens were lower in cress rotation under 80% SWC than under 100% SWC and the control (*P* < 0.05) (Figure 5).

### Effects of Total Soil Biota on Cucumber Seedling Growth

**FIGURE 6:**
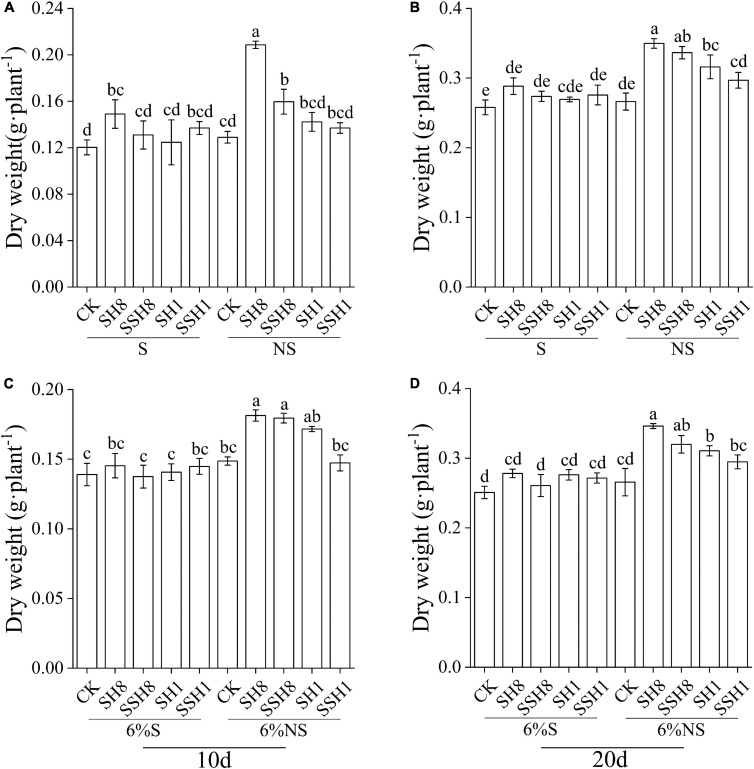
Effect of soil sterilization on cucumber seedling dry weight. **(A,B)** stand for soil sterilized (S) and non-sterilized (NS), respectively. **(C,D)** stand for 94% sterile background soil mix with 6% soil sterilized and 6% non-sterilized (NS). SH8, SSH8, SH1, and SSH1 represent single or double cress rotation under 80 or 100% soil water content.

Cucumber seedlings grown in the NS soil cress rotation under 80% SWC had a significantly higher dry biomass than in the S soil cress rotation under 80% SWC at 10 and 20 days, and the NS soil single cress rotation under 100% SWC had higher dry biomass compared with the S soil of the single cress rotation at 20 days (*P* < 0.05) (Figures 6A,B). Single cress rotation and cress rotation under 80% SWC had higher dry biomass than the control in NS soil (*P* < 0.05) (Figures 6A,B).

Cucumber seedlings grown in soil inoculated with the NS soil (6%) of the single cress rotation and cress rotation under 80% SWC had higher dry biomass than in the control and in soil inoculated with the S soil (6%) at 10 d and 20 d (*P* < 0.05) (Figures 6C,D).

## Discussion

Controlled SWC can enhance water use efficiency and increase crop yield (Song et al., 2021). As well as rotation can increase “temporary” plant diversity over short periods of time and reassemble the soil microbial community in the crop rhizosphere, which is also conducive to rhizosphere health and increase crop yields (Zhou et al., 2017, 2018b). Our study found that a single cress rotation and rotation under 80% SWC increased the cucumber yield. Research indicated that increasing precipitation and soil water availability can increase crop performance and the increase is related to the pattern of crop rotation (Wei et al., 2021; Xuan et al., 2012). Therefore, cress cultivated with varying degrees of flooding was rotated with cucumber could affect cucumber development.

Soil water management strategies can affect SWC as well as oxygenation levels, which in turn can mediate microbial diversity is likely to be different (Wang et al., 2014). Appropriated soil water management can balance both aerobic and anaerobic taxa and lead to the higher microbial diversity in the cucumber rhizosphere, as indicated by the higher microbial diversity in cress rotation under 80% SWC compare with cress rotation under 100% SWC and the control. Heterogeneity of the soil habitat can also increase microbial diversity (Shen et al., 2021), as well as SWC is the main factor affecting the diversity of microorganisms in the cucumber rhizosphere. These results had similar trends observed in most previous studies (Jiao et al., 2019). Higher diversity usually shapes more ecological functions (Nazaries et al., 2021), we can speculate that cress rotation under 80% SWC renders soil microorganisms and crop performance more “plastic.” The bacteria diversity in the cucumber rhizosphere was higher in single cress rotation than in double under 80% SWC, the opposite in fungi. This observation may be related to the abiotic factors, such as anaerobia degree of soil environment affects the difference of microorganism taxa (Kogel-Knabner et al., 2010). Bacteria and fungi in cucumber rhizosphere were respond differently to the degree of flooding.

Varying degrees of flooding can exert a selective pressure on the distribution of soil microbial communities, and microbial structure changes accordingly (Ali et al., 2019). Principal coordinate analysis demonstrated that the structure of soil microbial communities was separated in order of flooding duration and SWC along first and second axes, respectively. The structure of the soil microbial community produced different environmental niches due to flooding duration and SWC, such that microorganisms enriched their own unique community structure in the cucumber rhizosphere. This provides additional support for the argument that environmental changes lead to heterogeneous changes in the distribution of species in that community.

Interestingly, the diversity of soil microbial communities in double cress rotation under 100% was no different from control, and the structure is more similar closer to control. Generally, large-scale fluctuations in soil microorganism populations accumulate with extended periods of flooding and eventually result in changes in the microbial community. Obviously, our result did not support this argument and was contrary to our second hypothesis. The possible reason was soil microorganisms experience greater physiological stress in cress rotation under 100% SWC than in cress rotation under 80% SWC, which can result in the death of microorganisms or force them to enter a dormant stat (Che et al., 2020). The longer of flooding duration, the longer it will take for microorganisms to recover from the hypoxic stress caused by the flooding to achieve a higher respiratory intensity and activity rate than before (Lucía and Ana, 2015). This result explained that double cress rotation under 100% SWC may eliminate the difference in microbial diversity from dry land.

The soils in different habitats each have a unique community composition and OTU number. These unique dominant taxa shape the unique functions of this group. The relative abundances of *Mycobacterium*, *Kribbella*, *Streptomyces*, *Amycolatopsis*, and *Aeromicrobium* spp. were lower in double cress rotation under 80% SWC than in the control, and all of these taxa are members of Actinobacteria and are common in dry alkaline soils (Bhatti et al., 2017). Blame this on flooding reduces the oxygen flux in soil are suppressive the survival of Actinomycetes in double cress rotation, resulting in the reduction of bacterial functional genes related to metabolism in our result. Cress rotation under 100% SWC had higher relative abundance of Basidiomycota than the control. Zhao et al. (2018) showed that rainfall increased the numbers of Basidiomycota which is consistent with our result. The Zygomycota and *Mortierella* spp. which is benefits improve crop performance (Liu et al., 2020b) had higher relative abundance in cress rotation under 80% SWC than in the control; however, the Ascomycota and *Ilyonectria* spp. which is potential pathogens (Carron et al., 2020) had higher in control than in the other treatments. Meanwhile, the relative abundance of guilds related to plant pathogens had no changed in single cress rotation under 100%, instead, it was reduced in the cress rotation under 80%. This can be explained in the soil with higher diversity in the cress rotation under 80%. Thus, our study highlights the fact that differences in the soil environment can increase the heterogeneity in the spatial distribution of microorganisms and that ecological effects can be predicted from differences in soil environments.

The relative abundance of bacteria *Roseiflexus* spp. and fungi *Pseudallescheria* spp. were the highest in single cress rotation under 80% SWC then higher in double than in the control, that are known to potentially beneficial microbial, both of which enhance the absorption of nutrients in the rhizosphere by increasing competition to promote crop growth (Xu et al., 2012; Li et al., 2020; Shi et al., 2020; Zhu et al., 2020). Besides, the relative abundance of bacteria *Haliangium* spp. was higher and fungi *Fusarium* and *Monographella* spp. were lower in single cress rotation under 100% than in double and control. *Haliangium* spp., as biocontrol genera, can produce haliangicin to inhibit the growth of various fungi (Fudou et al., 2001) and fungi *Fusarium* and *Monographella* spp. were the potentially pathogenic fungi (Li et al., 2016; Zhu et al., 2019). The mechanism of the increase or decrease of potential beneficial microbial or pathogens due to varying degrees of flooding is still unclear, which is worthy of further discussion in the future.

Soil microorganisms can directly affect plant performance by forming symbiotic, parasitic or reciprocal relationships with plants (van der Heijden et al., 2008). The soil microbial communities induced by flooding were changes, however, what effect of soil microbial caused by varying degrees of flooding on cucumber seedling growth is unknown. We found that dry biomass of cucumber seedlings was increased in unsterilized soil of cress rotation under 80%, indicating that the microbial changes effect cucumber seedling growth. At 20 days, dry biomass also higher in unsterilized soil of single cress rotation under 100% than in sterilized soil of single cress rotation under 100%, may be due to slow-growing organisms such as Gemmatimonadetes were colonize microcosms lower than fast-growing organisms such as Firmicutes (Fierer et al., 2007; Bevivino et al., 2014). Therefore, single cress rotation under 100% also affects cucumber growth. On the other hand, dry biomass of cucumber seedlings was increased when grown in 6% unsterilized soil of cress rotation under 80% and single cress rotation. These results indicated that, single cress rotation and cress rotation under 80% can promote cucumber growth through inducing positive plant-soil microbial interaction.

## Conclusion

Overall, microbial community characters were significantly different when varying degrees of flooding were conducted, and yet only single cress rotation and cress rotation under 80% SWC increased cucumber yields. Regulating SWC under 80% of cress cultivation could thus be an efficient way to promote the colonization of potentially beneficial bacteria such as *Roseiflexus* and *Pseudallescheria* spp. It is also possible to reduce potential pathogenic fungal such as *Fusarium* and *Monographella* spp. in single cress rotation under 100% SWC. These results indicate that changing the heterogeneity of the soil-water environment in dryland is beneficial to increasing crop productivity via alterations in microorganisms; the mechanism underlying these effects requires further study. Additional work is needed to explore changes in plant performance mediated by different microbial mechanisms under different SWCs.

## Data Availability Statement

The sequence was normalized effort according to the samples that having minimum sequence number. The bacterial and fungal data sets were deposited in the NCBI-Sequence Read Archive with the submission Accession Numbers PRJNA644465 and PRJNA643002, respectively (https://www.ncbi.nlm.nih.gov/bioproject/PRJNA644465 and https://www.ncbi.nlm.nih.gov/bioproject/PRJNA643002).

## Author Contributions

XZ and FW conceived and designed the study. XZ and JS performed the experiments. XG analyzed the data and wrote the manuscript. TY and DG assist in perfecting thesis writing. All the authors have read and approved the final manuscript.

## Conflict of Interest

The authors declare that the research was conducted in the absence of any commercial or financial relationships that could be construed as a potential conflict of interest.

## Footnotes

1 http://www.arb-silva.de

2 http://unite.ut.ee/index.php

3 https://www.drive5.com/uchime/uchime_download.html
