# Supplementary material for: Crop Rotation With Cress Increases Cucumber Yields by Regulating the Composition of the Rhizosphere Soil Microbial Community
Source: Front Microbiol. 2021 Mar 12;12:631882. doi: 10.3389/fmicb.2021.631882 (PMC7994511; doi:10.3389/fmicb.2021.631882)
Supplement: Supplementary file 1 [file Table_1.doc]

Tab.S1 Effect of cress rotation on the α-diversity of soil bacterial and fungal communities in cucumber rhizosphere

| Types | Treatments | Number of OTUs | Chao 1 | ace | Shannon | Inverse Simpson | Coverage（%） |
| --- | --- | --- | --- | --- | --- | --- | --- |
|  | CK | 3164±48 b | 4388±119b | 4650±469 a | 6.61±0.01 d | 230.77±12.01 b | 96.92 |
|  | SH8 | 3280±124 b | 4332±45 b | 4478±76 a | 6.74±0.03 b | 334.64±16.97 ab | 97.03 |
| Bacterial | SSH8 | 3528±79 a | 4696±14 a | 4720±58 a | 6.84±0.01 a | 371.11±5.01 a | 97.15 |
|  | SH1 | 3341±95 ab | 4498±52 ab | 4541±26 a | 6.69±0.02 c | 292.07±94.19 ab | 97.15 |
|  | SSH1 | 3294±57 b | 4617±115a | 4574±45 a | 6.65±0.01 d | 303.71±52.73 ab | 97.08 |
|  | CK | 459±14 cd | 622±47 a | 607±36 ab | 2.42±0.06 c | 4.05±0.27 b | 99.78 |
|  | SH8 | 513±15 ab | 648±35 a | 650±32 a | 3.11±0.11 b | 9.12±0.72 ab | 99.79 |
| Fungal | SSH8 | 553±15 a | 664±45 a | 674±37 a | 3.34±0.07 a | 10.28±0.71 a | 99.79 |
|  | SH1 | 491±12 bc | 613±22 a | 634±28 ab | 2.63±0.07 c | 4.54±0.20 b | 99.78 |
|  | SSH1 | 443±20 d | 571±70 a | 563±39 b | 2.54±0.09 c | 5.21±0.38 b | 99.81 |

Tab.S2 Soil bacterial function prediction of cucumber rhizosphere in different rotation treatment

(Hierarchy level 2)

|  | CK | SH8 | SSH8 | SH1 | SSH1 |
| --- | --- | --- | --- | --- | --- |
| Biosynthesis of other secondary metabolites | 1.04±0.0032b | 1.04±0.0028b | 0.88±0.0031c | 1.05±0.0011a | 1.03±0.0022c |
| Infectious disease: bacterial | 1.20±0.012b | 1.18±0.0052c | 1.05±0.0070a | 1.24±0.0078a | 1.25±0.0081a |
| Cell growth and death | 1.58±0.00041d | 1.59±0.0029cd | 1.36±0.0048a | 1.60±0.0071c | 1.61±0.0011b |
| Cell motility | 1.81±0.023c | 1.86±0.024b | 1.59±0.015a | 1.91±0.013b | 1.87±0.0077b |
| Folding, sorting and degradation | 2.33±0.0047a | 2.29±0.0093b | 1.97±0.011a | 2.32±0.011a | 2.33±0.0046a |
| Glycan biosynthesis and metabolism | 2.52±0.0048ab | 2.47±0.012b | 2.14±0.016a | 2.51±0.032 b | 2.51±0.010 b |
| Metabolism of other amino acids | 2.67±0.0028a | 2.66±0.000080a | 2.25±0.0092b | 2.65±0.0097a | 2.66±0.0032a |
| Metabolism of terpenoids and polyketides | 3.86±0.013a | 3.83±0.025ab | 3.21±0.035c | 3.88±0.026a | 3.77±0.035bc |
